# Ligand engineering to achieve enhanced ratiometric oxygen sensing in a silver cluster-based metal-organic framework

**DOI:** 10.1038/s41467-020-17200-w

**Published:** 2020-07-22

**Authors:** Xi-Yan Dong, Yubing Si, Jin-Sen Yang, Chong Zhang, Zhen Han, Peng Luo, Zhao-Yang Wang, Shuang-Quan Zang, Thomas C. W. Mak

**Affiliations:** 1 Green Catalysis Center, and College of Chemistry, Zhengzhou University, 450001 Zhengzhou, China; 2 College of Chemistry and Chemical Engineering, Henan Polytechnic University, 454003 Jiaozuo, China; 3 Department of Chemistry, The Chinese University of Hong Kong, Shatin, New Territories, Hong Kong SAR, China

**Keywords:** Chemical synthesis, Coordination chemistry

## Abstract

Ratiometric luminescent oxygen sensing based on dual fluorescence and phosphorescence emission in a single matrix is highly desirable, yet the designed synthesis remains challenging. Silver-chalcogenolate-cluster-based metal-organic frameworks that combine the advantages of silver clusters and metal-organic frameworks have displayed unique luminescent properties. Herein, we rationally introduce −NH_2_ groups on the linkers of a silver-chalcogenolate-cluster-based metal-organic framework (Ag_12_bpy-NH_2_) to tune the intersystem crossing, achieving a dual fluorescence-phosphorescence emission from the same linker chromophore. The blue fluorescence component has a 100-nm gap in wavelength and 8,500,000-fold difference in lifetime relative to a yellow phosphorescence component. Ag_12_bpy-NH_2_ quantifies oxygen during hypoxia with the limit of detection of as low as 0.1 ppm and 0.3 s response time, which is visualized by the naked eye. Our work shows that metal cluster-based MOFs have great potential in luminescent sensing, and the longer-lived charge-separated states could find more photofunctional applications in solar energy transformation and photocatalysis.

## Abstract (web summary)

Incorporating dual fluorescence and phosphorescence into a single matrix is attractive for oxygen sensing, but material design is challenging. Here the authors achieve dual fluorescence-phosphorescence from a single linker chromophore in a silver chalcogenolate-cluster-based metal-organic framework.

## Introduction

**Fig. 1:**
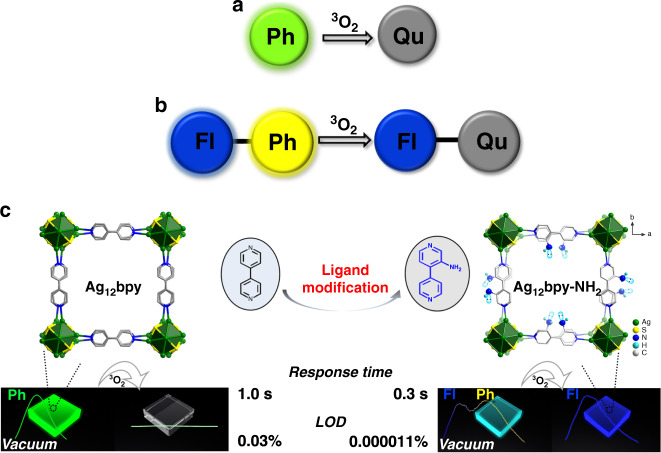
Schematic luminescent oxygen sensing and ligand modification of SCC-MOFs. Schematic of oxygen quenching sensing based on **a** a single Ph emission and **b** dual Fl-Ph emissions. Fl = Fluorescence, Ph = Phosphorescence, Qu = Quenched. Each colored circle represents an emissive center. **c** Schematic of Ag_12_bpy crystals emitting a single green color under vacuum that is quenched by oxygen with a response time of approximately 1.0 s and an LOD of 0.03%; the isostructural Ag_12_bpy-NH_2_ crystal modified with −NH_2_ groups on the bpy linkers emits nearly a different cyan color that is composed of a blue Fl and a yellow Ph component under a certain vacuum, but only emits blue Fl under oxygen conditions. Note: the color emitted by Ag_12_bpy-NH_2_ is variable under different vacuum conditions (see the main text). When using Ag_12_bpy-NH_2_ as a ratiometric oxygen sensor, the response time decreased to 0.3 s and the LOD dropped to 0.000011%.

Oxygen quantification during hypoxia is essential in many fields of science and technology^1–3^, and luminescent O_2_ sensing has unique advantages, including full reversibility and a good precision and accuracy^2,3^. Commonly, the phosphorescence (Ph) emission intensity and lifetime are easily quenched to a certain extent by triplet oxygen, which becomes the basis of luminescent sensors used to detect molecular oxygen (Fig. 1a)^2–7^. However, it remains challenging to obtain an ultrahigh sensitivity with minimum system error and to detect trace amounts of O_2_. Scientists are pursuing self-calibrating ratiometric fluorescence (Fl)-phosphorescence (Ph) dual emission in a single luminescent matrix^8–12^ with a longer-lived Ph component^13^ (Fig. 1b), which can not only avoid the errors that are induced by the stoichiometric imbalance of the different emitting centers but also exhibit easily perceived color changes for rapid visual sensing (Fig. 1b). However, thus far, designing such integrated luminophores has achieved limited success for the following reasons: first, achieving balanced Fl and Ph intensities originating from the same luminophore is challenging for a ratiometric O_2_ sensor because, as stated by Kasha’s rule, photon emissions occur from only the lowest excited state^14^; second, phosphors that have both long-lived Ph (over milliseconds) and a high quantum yield (QY) at room temperature are difficult to prepare due to the intrinsic competition between the Ph lifetime and efficiency^15^; third, this type of luminophore must have a high oxygen permeability for free gas diffusion^2–8^.

Metal-organic frameworks (MOFs) feature flexible secondary-building units (SBUs) and infinite tunable organic linkers, as well as abundant and distinctive pores or channels, and hence have shown great potential for separation and sensing^8–11,16–32^. Traditionally, dual emission in MOFs is mostly achieved with lanthanide ion or luminescent linker on a host MOF framework as one emissive center and a post-introduced luminescent species including lanthanide ion, dye or carbon dot as another emissive center, which make it difficult to realize ultrasensitive and ratiometric detection towards oxygen gas^11^. Silver-chalcogenolate-cluster-based metal-organic frameworks (SCC-MOFs)^33–38^_,_ in which each SBU consists of tens of silver and chalcogen atoms, have become a new member of the MOF family. SCC-MOFs combine the advantages of silver clusters^39–43^ and MOFs^8–11,16–32^; SCC-MOFs have shown remarkably improved luminescent properties relative to discrete silver clusters and unique excellent luminescent responses for sensing gases and volatile organic compounds (VOCs)^33–37^. We previously reported a SCC-MOF, that is, Ag_12_bpy^33^, ([Ag_12_(S^t^Bu)_8_(CF_3_COO)_4_(bpy)_4_]_n_, bpy = 4,4′-bipyridine), whose single green Ph is sensitive to oxygen (Fig. 1c). We hypothesize that, according to the interligand trans-metallic charge-transfer (ITCT) emission mechanism of Ag_12_bpy^33,36^, the additional introduction of the organic ligand-centered Fl emission can achieve dual Fl-Ph emission centers in the host framework of a SCC-MOF, whose Ph component is supposed to be sensitive to oxygen; the functional modification of the MOF linker can modulate the Ph properties by changing the intersystem crossing (ISC) efficiency; thus, ratiometric oxygen sensing could be achieved in a SCC-MOF with a higher sensitivity.

**Fig. 2:**
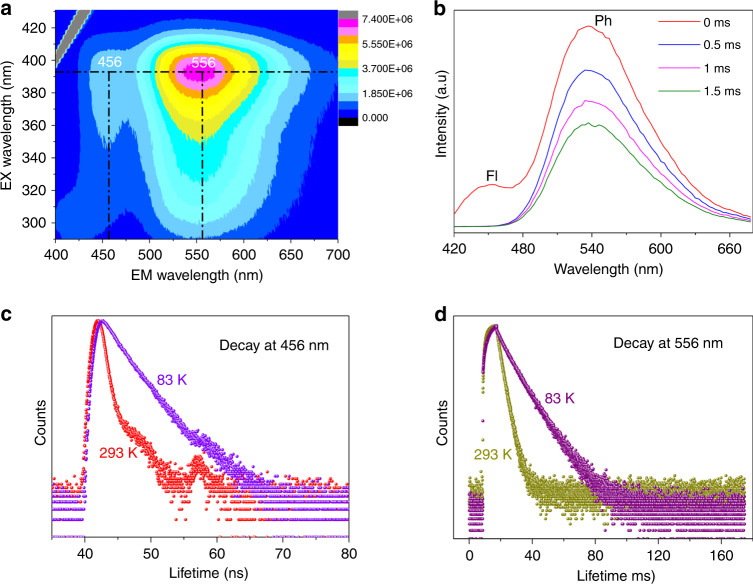
Photoluminescence properties of Ag_12_bpy-NH_2_. **a** The three-dimensional excitation-emission matrix (3D-EEM) spectra of Ag_12_bpy-NH_2_ under a vacuum at room temperature. The two emission centers are located at ~456 and 556 nm. **b** Prompt (0 ms) and time-delayed (0.5, 1, 1.5 ms) emission of solid-state Ag_12_bpy-NH_2_ at room temperature under a vacuum. **c** Time-resolved Fl (456 nm) and **d** Ph (556 nm) decay traces of Ag_12_bpy-NH_2_ under a vacuum at 293 and 83 K.

Here, we first modified the bpy linkers using amino groups bearing lone-pair electrons, i.e., a blue emitting 3-amino-4,4′-bipyridine (bpy-NH_2_)^31,36^ moiety, and changed the synthesis method (Supplementary Fig. 1), yielding a new isostructural complex, [Ag_12_(SBu^t^)_8_(CF_3_COO)_4_(bpy-NH_2_)_4_]_n_ (Ag_12_bpy-NH_2_) (Fig. 1c). The incorporation of amino groups has two significant functions: first, a blue sub-nanosecond Fl component (0.37 ns under vacuum) centered at ~456 nm (Figs. 1c and 2), in addition to a yellow Ph component at 556 nm, has been achieved; second, incorporating amino groups strongly triggers spin-orbit coupling (SOC) and increases the rate of ISC to boost triplet excitons, thus prolonging the lifetime of the Ph component by ~15,000-fold, from submicroseconds (0.2 µs) to milliseconds (3.12 ms), which occurs concomitantly with an increase in the QY from 12.1 to 14.6% under vacuum. Thus, the large gap of ~100 nm between the emission wavelengths and the increased lifetime difference (~8.5 × 10^6^ times) of the Ph and Fl bands, combined with the highly oxygen-permeable framework structure, enable Ag_12_bpy-NH_2_ to function as a single-component Fl-Ph ratiometric sensor for molecular oxygen in which ultrafast responses towards trace oxygen gas are achieved in 0.3 s, and the limit of detection (LOD) is as low as 0.1 ppm; this response can be visualized by variations in the color, which is dependent on the O_2_ concentration below 20 ppm. To the best of our knowledge, Ag_12_bpy-NH_2_ is the first report of a Fl-Ph dual-emissive MOF that has a record-breaking LOD and response speed^8–10,19–25^. By the further introduction of bulk −CH_3_ groups on the partial bpy linkers, which interferes with the oxygen collision dynamics, we then prepared another isostructural mixed-linker Ag_12_bpy-NH_2_/CH_3_, which extended the ratiometric sensing range. Such long-lived metal-cluster ensembles are promising materials for sensing, photocatalysis, sensitization of electrochemical solar cells, and solar-energy-harvesting applications.

## Results

### Structure and photoluminescence of Ag_12_bpy-NH_2_

Compared to bpy, bpy-NH_2_ has an unchanged length and unaltered connectivity (Fig. 1c); however, introducing an amino group at the 3-position of bpy greatly alters the reaction conditions: the synthesis method used for the Ag_12_bpy structures that are synthesized in mixed CH_3_CN and C_2_H_5_OH is no longer applicable for Ag_12_bpy-NH_2_. After sustained attempts, isomorphous single crystals of Ag_12_bpy-NH_2_ were prepared by the reaction of AgS^t^Bu, CF_3_COOAg and bpy-NH_2_ in a mixed solvent of NH_4_OH and CH_2_Cl_2_ (Supplementary Fig. 1), suggesting that the used solvents play key roles in the structure of SCC-MOFs. The single-crystal X-ray diffraction (SCXRD) analysis revealed that Ag_12_bpy-NH_2_ and Ag_12_bpy belong to the same tetragonal space group (No. 121) and have identical silver cluster subunits, and every (−S^t^Bu, −OOCCF_3_) ligand and N-containing linker have identical ligand coordination modes (Fig. 1c and Supplementary Figs. 2 and 3). The phase purity and chemical formula of Ag_12_bpy-NH_2_ were further verified by elemental analysis, powder X-ray diffraction (PXRD) and thermogravimetric analysis (TGA) (Supplementary Figs. 4 and 5). The amino groups anchored on the linker slightly decrease the pore void space from 21.6% for Ag_12_bpy to 17.5% for Ag_12_bpy-NH_2_, as calculated from the X-ray structural data obtained by PLATON (see Methods) and verified by the measurement of the 77 K N_2_ sorption isotherm (Supplementary Fig. 6). Compared to that of Ag_12_bpy, the UV-vis absorption intensity of Ag_12_bpy-NH_2_ obviously increased before 600 nm and redshifted, extending to 680 nm (Supplementary Fig. 7). The enhancement before 450 nm suggests that new (n, π*) transitions possibly occurred because of the introduction of the −NH_2_ group with a lone-pair on the pyridine ring (Fig. 1c, Supplementary Figs. 8 and 9).

The crystals of Ag_12_bpy-NH_2_ exhibit a blue emission in air and in solution not degassed by N_2_, which is similar to the blue Fl color of the pure bpy-NH_2_ linker^36^ in the solid state with a lifetime of approximately 3.59 ns (Table 1 and Supplementary Figs. 10–12). In contrast, amino-free Ag_12_bpy exhibits color-variable Ph in various organic solvents^33^. These observations implied that the Ph component of Ag_12_bpy-NH_2_ is likely to be much more sensitive to oxygen in solution than Ag_12_bpy. To validate this hypothesis, we subsequently measured the luminescence properties of Ag_12_bpy-NH_2_ under a high vacuum at room temperature, which resulted in highly separated dual emissions (Fig. 2a, b and Table 1) upon excitation with a 370 nm UV light. One emission is the ligand-based fluorescence emission centered at ~456 nm, which has a lifetime of 0.37 ns at room temperature and 2.48 ns at 83 K (Fig. 2c); this emission lifetime of 0.37 ns is only one-tenth the lifetime of the solid-state bpy-NH_2_ ligand (3.59 ns, Table 1 and Supplementary Fig. 11); the Fl QY is significantly reduced from 30.56 to 2.54%, and the Fl peak is also redshifted to 456 nm compared to the Fl peak of solid-state bpy-NH_2_ (420 nm) due to metal coordination (Supplementary Fig. 10). Another emission is an outstanding yellow Ph emission located at 556 nm, with emission lifetimes of 3.12 ms at 293 K and 10.55 ms at 83 K (Fig. 2d), which is an increase of 15,000 times compared to the room-temperature Ph lifetime of 200 ns observed for unsubstituted Ag_12_bpy; in addition, the quantum efficiency increased to 14.62% compared to the 12.10% quantum efficiency obtained for unsubstituted Ag_12_bpy under vacuum (Table 1). Most importantly, the separation of the Ph peak at 556 nm from its Fl peak at 456 nm is truly enhanced, and the Ph intensity greatly surpasses the Fl intensity under vacuum (Fig. 2b). The significant decrease in the ligand-based Fl lifetimes of Ag_12_bpy-NH_2_ from 3.59 to 0.37 ns, which is accompanied by a considerable increase in the Ph lifetime from 0.2 μs to 3.12 ms (Table 1), suggested the occurrence of an efficient ISC process from the lowest singlet to the lowest triplet excited states (vide infra). The 15,000-fold enhancement in the Ph lifetime is an unprecedented phenomenon among luminescent MOFs^19–28,33–38^ and metal clusters^39–43^, even in metal-containing phosphors^12,13,44–46^, which means that the triplet excited state(s) of Ag_12_bpy-NH_2_ is capable of responding to oxygen molecules with a sensitivity of one-15,000th of the original sensitivity of unsubstituted Ag_12_bpy if one merely considers the lifetime factor. Of course, this also presents a challenge for obtaining precise measurements in the range of hypoxia.

**Table 1:** Luminescent properties of the compounds.

Compound	Fluorescence			Phosphorescence		
	λ_max_ (nm)	τ_f_ (ns)	QY(%)	λ_max_ (nm)	τ_p_(ms)	QY(%)
bpy-NH_2_	420	3.59	30.56	No	No	No
Ag_12_bpy	No	No	No	507	2 × 10^−4^	12.10
Ag_12_bpy-NH_2_	456	0.37	2.54	556	3.12	14.62
Ag_12_bpy-CH_3_	No	No	No	500	6.8 × 10^−5^	3.06
Ag_12_bpy-F	No	No	No	530	3.5 × 10^−4^	21.80

*λ*_*max*_ *(nm)* the maximum of emission wavelength, *τ*_*f*_ the lifetime of fluorescence component, *τ*_*p*_ the lifetime of phosphorescence component, *QY* quantum yield.

Unless otherwise stated, at 20 °C under vacuum.

### Ratiometric optical oxygen sensing

By balancing the Fl and Ph intensities upon excitation at a single wavelength in which the Ph component has a considerably long decay time (3.12 ms) and highly separated dual emissions, as well as highly oxygen-permeable channels (Figs. 1, 2 and Table 1), Ag_12_bpy-NH_2_ provides a platform to serve as a ratiometric O_2_ sensor and is ideal for the characterization of nearly anoxic systems. Because of the ultrasensitive oxygen quenching effect of Ag_12_bpy-NH_2_, measurements must be carried out in a high vacuum system because an inert atmosphere (less than 10 ppb of oxygen) is very difficult to achieve for standard O_2_ gas sensing. To overcome the difficulty of the measurement, we used a homemade measurement system in conjunction with a gas absorption analyzer (BEL-max physisorption analyzer) to ensure the control of the ultrahigh vacuum with high precision and a spectrofluorometer (HORIBA FluoroLog-3) that was connected to a gas absorption analyzer (MicrotracBEL Belsorp Max) by an optical fiber. An image of this set up for O_2_ sensing under an ultrahigh vacuum is shown in Supplementary Fig. 13 with a detailed explanation.

**Fig. 3:**
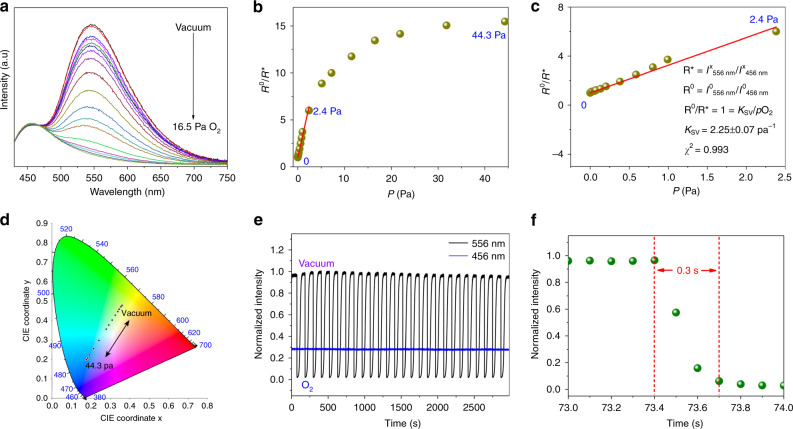
Luminescent oxygen sensing. **a** Emission spectra of Ag_12_bpy-NH_2_ under different oxygen pressures from 0 to 16.5 Pa (excited at 365 nm). **b** The correlation between the ratiometric photoluminescence response and O_2_ partial pressure in the range of 0–44.3 Pa. **c** Representative Stern-Volmer plot of O_2_ in the range of 0–2.4 Pa. **d** The emission color-changing range for ratiometric oxygen sensing. **e** Reversible luminescence cycles of Ag_12_bpy-NH_2_ under alternating exposure to air/vacuum: on-off Ph (black folding line) and invariant Fl (blue straight line). **f** Enlargement of the response transient curves of Ag_12_bpy-NH_2_ exposed to air. The response time of the air-quenching Ph component is ~0.3 s (the response time is defined as the time corresponding to a 90% decrease in the emission intensity when the gas phase is changed from vacuum to air).

Ratiometric optical oxygen sensing using dual-emitting Ag_12_bpy-NH_2_ was achieved by monitoring the response at different oxygen partial pressures (Fig. 3a, b). Oxygen-insensitive blue Fl served as the ‘reference’ signal, and oxygen-quenchable Ph served as the response signal for readout. The ratio of the nearly invariant fluorescence response (λ_Fl_ = 456 nm) to the oxygen-dependent Ph response (λ_Ph_ = 556 nm) steadily increased with increasing oxygen levels. Overall, 99.9% of the phosphorescence response was quenched at a pressure of 44.3 Pa. In the range from vacuum (10^–3^ Pa) to 2.4 Pa, a linear Stern-Volmer plot (SVP)^2,8,33^ was observed (*R*^2^ = 0.993) (Fig. 3c), and a continuous linear color change from blue to yellow was observed in the CIE coordinate diagram, facilitating the identification of the O_2_ pressure by the naked eye (Fig. 3d). The calculated Stern-Volmer constant^2,8,33^ (*K*_sv_) was 2.25 kPa^−1^, and the LOD at 1% quenching was 11.4 mPa. Beyond this point, the Fl/Ph plot continued to increase up to 44.3 Pa, which was nearly equal to ambient levels (that is, 21% O_2_) (Fig. 3b). Compared to the LOD (32 Pa) of Ag_12_bpy, the LOD of Ag_12_bpy-NH_2_ was low (11.4 mPa), only 3.56 × 10^−6^% of the original value. To further explore the oxygen sensitivity, quick response and recyclability of this system, we recorded the transient luminescence response curves of the dual-emission peak intensity under alternating air/vacuum conditions at RT (Fig. 3e); the nearly invariant Fl intensity at 456 nm (blue straight line) and the on-off Ph intensity with sharp contrast at 556 nm (black folding line) demonstrated that Fl is a good reference signal. The air-quenching response time was 0.3 s (Fig. 3f), which is as fast 3.3 times as that of Ag_12_bpy (~1 s)^33^, and is concomitant with the synchronous visual color change (Fig. 3d). Compared to the Ph lifetime of Ag_12_bpy (0.2 µs)^33^, we think that the elongated Ph lifetime of Ag_12_bpy-NH_2_ of 3.12 ms mainly contributes to the enhanced sensitivity. The calculated void space of Ag_12_bpy-NH_2_ was 17.5%, which is slightly lower than that of Ag_12_bpy (21.6%), as demonstrated by the 77 K N_2_ absorption isotherm (Supplementary Fig. 6). Ag_12_bpy-NH_2_, with an LOD as low as 0.11 ppm, is applicable for nearly anoxic systems with oxygen levels in the range of 0.1–24 ppm and has sensitivities that are orders of magnitude above those of conventional sensing MOFs^19–28,33–38^. Ag_12_bpy-NH_2_ opens the door to completely new applications for monitoring oxygen in previously inaccessible concentration regions and is likely to become invaluable in diverse fields of science and technology.

### Extending the sensing range of O_2_ by introducing bulky −CH_3_ groups

**Fig. 4:**
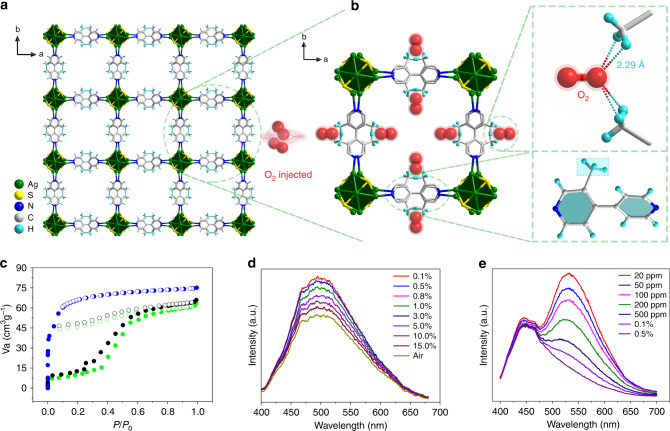
The oxygen molecule position in Ag_12_bpy-CH_3_ and the oxygen-sensing performance. **a** Channels of Ag_12_bpy-CH_3_ and **b** O_2_-inclusion of Ag_12_bpy-CH_3_·O_2_, as viewed along the *c*-axis. The most preferable positions of the oxygen molecules are displayed. The smallest separation between O_2_ and the − CH_3_ groups (O···H) is equal to 2.29 Å. Color code: Ag, green; S, yellow; C, gray; N, blue; H, light turquoise; O, red. The H atoms of bpy and −^t^Bu groups of the host backbone are omitted for clarity. **c** N_2_ adsorption/desorption isotherms of Ag_12_bpy-NH_2_ (blue line), Ag_12_bpy-CH_3_ (green line) and Ag_12_bpy-NH_2_/CH_3_ (1:100) (black line) at 77 K. **d** Oxygen-dependent emission intensity of Ag_12_bpy-CH_3_ in the range of 0.1% to ambient air conditions. **e** Oxygen-dependent dual-emission intensity of Ag_12_bpy-NH_2_/CH_3_ (1:100) in the range of 20 ppm to 0.5%.

Considering the dynamic O_2_-quenching mechanisms of these SCC-MOF_S_, one can imagine that other factors that affect the oxygen collision process would also tune the sensitivity. Based on this hypothesis, we prepared another isostructural SCC-MOF, Ag_12_bpy-CH_3_ (Fig. 4a, b), where the large methyl group, which contributes minimally to the excited state, is expected to intervene in the collision process (Supplementary Figs. 14–19). In contrast to Ag_12_bpy-NH_2_, an opening effect and desorption lag are observed in the absorption isotherms of Ag_12_bpy-CH_3_ at 77 K (Fig. 4c), and a decrease in the pore volume also appeared due to the relatively increased size of the −CH_3_ groups, suggesting that the possible weakly viscous interactions between the host and guest hindered gas from freely going in and out. Ag_12_bpy-CH_3_ merely emits a single green luminescence response (Fig. 4d, Table 1) under vacuum and weakly luminesces in air, implying that the Ph emission of Ag_12_bpy-CH_3_ is much less sensitive to oxygen than that of Ag_12_bpy-NH_2_. The oxygen-sensing experiments showed that Ag_12_bpy-CH_3_ can work in the range of higher oxygen concentrations (0.1–21% in air, Fig. 4d). To further understand the origin of the insensitivity induced by the −CH_3_ groups, we prepared a molecular oxygen inclusion compound, namely, Ag_12_bpy-CH_3_·O_2_, at 80 K (Fig. 4b); the channels of Ag_12_bpy-CH_3_·O_2_ contained the most preferred position of O_2_ molecules, which had a site occupancy of 0.1 and were the nearby −CH_3_ groups, and the separation between the O_2_ and −CH_3_ groups (O···H = 2.29 Å, O···C = 2.65 Å) was much smaller than that between the O_2_ and bpy moieties (4.05 Å) (Supplementary Fig. 20). Considering that the kinetic diameter of O_2_ is 3.46 Å^32^ and that the −CH_3_ groups are minimally involved in the excited states, we proposed that O_2_ molecules have fewer chances to dynamically collide with LUMO localized bpy-CH_3_ in Ag_12_bpy-CH_3_ and can not effectively switch off their emission, resulting in insensitivity. Then, further than that, a mixed-linker crystal of Ag_12_bpy-NH_2_/CH_3_ (with a bpy-NH_2_:bpy-CH_3_ ratio of 1:100, as determined by ^1^H NMR, Supplementary Figs. 21 and 22) was prepared and functions in the O_2_ concentration range of 20 ppm to 0.1% (Fig. 4e). The contrasting effect of the −NH_2_ and −CH_3_ groups on the oxygen-sensing performance indicate that the proposed dynamic quenching process by oxygen is a photophysical process, during which the interactions between molecular (triplet) oxygen and the excited electronic states of the SCC-MOFs are controlled by the emissive lifetime and the permeability or diffusion rate of oxygen. The −NH_2_ groups, markedly enhanced the sensitivity by generating an oxygen-insensitive reference blue Fl signal and simultaneously elongating the yellow Ph component lifetime; while, the −CH_3_ groups mainly decrease the sensitivity by erecting barriers of O_2_ diffusion in the channels of the SCC-MOFs. The balance modulation can tune the sensing range of oxygen. Therefore, engineering the ligands on SCC-MOFs is deemed to be a powerful method of modulating the remarkable luminescent sensing functionality.

### Luminescent properties of isostructural SCC-MOFs with different substituents

To gain further insights into the origin of the dual emission and the long-lived Ph lifetime of Ag_12_bpy-NH_2_, we compared two additional isostructural crystals, Ag_12_bpy-CH_3_ containing bpy-CH_3_ linkers with an electron-donating methyl group and Ag_12_bpy-F^36^ containing 3-fluorine-4,4′-bipyridine (bpy-F) bearing an electron-withdrawing fluoro group (Table 1). Based on the above mentioned experimental results, we have observed that in the absence of an efficient Fl emission produced by bpy-CH_3_ and bpy-F, only a single phosphorescence response appears in Ag_12_bpy-CH_3_ and Ag_12_bpy-F. Similar to most metal-containing phosphors involving a π*-orbital-involved charge-transfer emission^12,44,45^, electron-donating −CH_3_ groups likely destabilize the π* orbital, decrease the lifetime to approximately 68 ns under vacuum, and cause a blueshift of only 7 nm under vacuum; however, electron-withdrawing −F groups have the opposite effect and increase the lifetimes (351 ns^36^, Table 1) and result in a redshift of more than 20 nm. This variation induced by the electron-donating/withdrawing effect still occurred on the same order of magnitude of submicroseconds, indicating that the inherent emissive excited state did not change. In stark contrast, the Ph component of Ag_12_bpy-NH_2_ unprecedentedly increased drastically by over four orders of magnitude. We proposed that the −NH_2_ substituent might take part in the emissive triplet state and promote ISC from the singlet to triplet state. The Fl component of Ag_12_bpy-NH_2_, with a lifetime of 0.37 ns, possibly originates from the mixed ^1^(n, π*) and ^1^(π, π*) excited singlet states; the Ph component of Ag_12_bpy-NH_2_ with a lifetime of 3.12 ms, might originate from the mixed ^3^ITCT and ^3^(n, π*) triplet states in which the participation of ^3^(n, π*) could facilitate ISC and probably varies the characteristics of the lowest emissive triplet states, eventually leading to a 15,000-fold elongation of the lifetime and a more than 40-nm redshift. The incomplete electron communication between the two emissive states could result in a Fl-Ph dual emission (Supplementary Fig. 23).

### The amino groups modulating the emissive triplet states and prompting ISC

The Fl energy of the free bpy-NH_2_ ligand is dependent on the solvent (Supplementary Fig. 10), which is consistent with the charge-transfer (CT) characteristics and further indicates that the emissive singlet state could combine the ^1^(n, π*) state with the ^1^(π, π*) state. In contrast, the Fl component of Ag_12_bpy-NH_2_ is independent of the solvent (Supplementary Fig. 12); it is likely that the ligand-based ^1^(π, π*) state may be the localized excited state at bpy-NH_2_ linkers of Ag_12_bpy-NH_2_ (Supplementary Fig. 23). From these comparison data, a schematic of the evolving luminescence is presented: the blue Fl component of the ligand, which stems from the emissive singlet state, was considerably decreased by 92.8%, which is calculated from the lifetime. For Ag_12_bpy, we previously ascribed the Ph component to ITCT (S/Ag → bpy)^36^. Thus, the Ph component of Ag_12_bpy-NH_2_ from the *ITCT triplet state (S/Ag → bpy-NH_2_) redshifted to 556 nm, and its intensity increased; the most attractive feature of this system is the increase in the lifetime to the millisecond range. The trend between Fl and Ph indicates that (i) the emissive triplet state of Ag_12_bpy-NH_2_ has a lower energy and is relatively stable, and (ii) the charge transfer and energy transfer from the bpy-NH_2_-based ^1^(π, π*)-dominated emissive single state to the ^3^ITCT (S/Ag → π* of bpy-NH_2_) triplet state was mixed with a small amount of ^3^(n, π*). Therefore, incorporating the −NH_2_ group into the bpy linkers significantly changed molecular energy levels (see the calculated molecular orbitals and analysis of Ag_12_bpy-NH_2_ shown in Supplementary Figs. 23–26 and Supplementary Table 1).

**Fig. 5:**
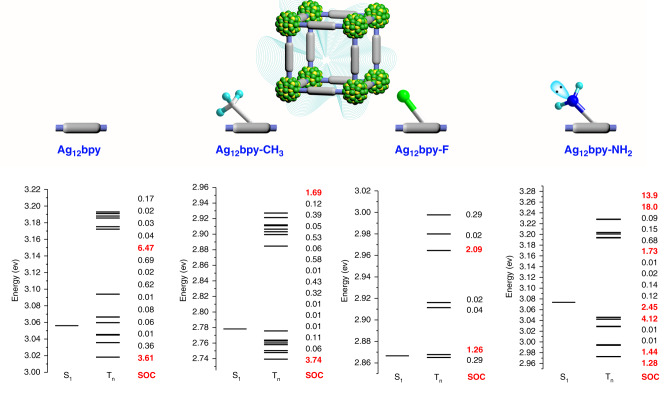
Energy level diagram and SOC constants. The SOC values are in cm^−1^, and the high values (>1 cm^−1^) are in bold red. There are 4, 8, 1, and 10 triplet states that are lower than the corresponding singlet state of the pristine, −CH_3_, −F, and −NH_2_ substituted molecules. Considering that the resonance structures may promote ISC^47^, the higher triplet states with a low energy gap (∆E_S1Tn_ < 0.15 eV) were displayed.

Moreover, because the rate constant of the ISC from singlet to triplet excited states is proportional to the square of the SOC constant, we examined 20 of the lowest singlet-triplet transitions by performing calculations for Ag_12_bpy-NH_2_, Ag_12_bpy-CH_3_, Ag_12_bpy-F, and Ag_12_bpy (Fig. 5). Considering that spin-flipping may be enhanced by the resonance variation with an increased n-value orbital participation^47^, the triplet excited states with a small vertical excitation energy gap (∆E_S1Tn_ less than 0.15 eV) were included. Ag_12_bpy-NH_2_ has far more triplet excited states (T_n_) close to S_1_ in energy, and the smaller energy gap and higher SOC would well support the efficient ISC process to generate more triplet states with much longer lifetimes. For Ag_12_bpy-NH_2_, the transitions between S_1_ and T_n_ are the transitions from locally excited (LE) states ^1^(π, π*) to charge-transfer triplet states mixed with ^3^(n, π*) and ^3^ITCT, which would result in the high SOC according to the El-Sayed rules^48–50^. Thus, the participation of the −NH_2_ units actually results in a new facile pathway for the ISC process to occur between S_1_ and T_n_, increasing the effective ISC to achieve an efficient longer-lived Ph response.

## Discussion

In summary, we have devised a simple yet powerful strategy to tune the delicate balance between the emissive intraligand singlet states and CT triplet states, which enables the observation of dual Fl and Ph peaks with a 100-nm separation and an 8,500,000-fold lifetime gap, and have provided the first model of dual Fl-Ph ultrasensitive ratiometric O_2_ sensing in the range of hypoxia based on a single-component SCC-MOF. The introduced amine groups (−NH_2_) with lone-pair electrons strengthened SOC and facilitated ISC and, henceforth, induced a 15,000-fold enhancement in the Ph lifetime from submicroseconds to milliseconds. Furthermore, using a substituent-mediated strategy (−CH_3_ and mixed −NH_2_ and −CH_3_ groups) to interfere with the dynamic quenching process of O_2_ extends the sensing concentration range. The bright long-lifetime excited states and the homogeneously ordered spatial separation of metal clusters enable these metal-cluster ensembles to be used in sensing, imaging, photocatalysis, and solar-energy-harvesting applications.

## Methods

### Reagents

All reagents and solvents used were of commercially available reagent grade and used without further purification. AgS^t^Bu was prepared from the reaction of molar equivalents of Ag_2_O and HS^t^Bu in Et_3_N.

### Synthesis of bpy-NH_2_ and bpy-CH_3_

3-amino-4,4′-bipyridine (bpy-NH_2_) and 3-methyl-4,4′-bipyridine (bpy-CH_3_) were prepared from Suzuki coupling of 3-amino-4-chloropyridine or 3-methyl-4-chloropyridine and pyridine-4-boronic acid^31^.

### Preparation of Ag_12_bpy-NH_2_ single crystals

3-amino-4,4′-bipyridine (bpy-NH_2_, 10 mg) and 10 µL ammonium hydroxide were added to a solution of AgS^t^Bu (15 mg), CF_3_COOAg (6 mg) and CH_2_Cl_2_ (4 mL) under stirring to obtain a clear solution under ambient conditions. The reaction solution was filtered, and the filtrate was slowly evaporated in air to give bulk colourless crystals, which were rinsed with CH_2_Cl_2_, filtered and dried in air to produce Ag_12_bpy-NH_2_ (C_80_H_108_Ag_12_F_12_N_12_O_8_S_8_) in ~30% yield based on Ag. Elemental analysis (calculated): C 30.55, H 3.46, N 5.34, S, 8.16%; Found: C 30.42, H 3.53, N 5.41, S, 8.09%.

### Preparation of Ag_12_bpy-CH_3_ single crystals

Single crystals of Ag_12_bpy-CH_3_ were prepared in a manner like the preparation of Ag_12_bpy-NH_2_, except bpy-NH_2_ was replaced by bpy-CH_3_; a yield of ~55% was achieved based on Ag. Ag_12_bpy-CH_3_ (C_84_H_112_Ag_12_F_12_N_8_O_8_S_8_) Elemental analysis (calculated): C 32.12, H 3.59, N 3.57, S, 8.17%; Found: C 32.18, H 3.64, N 3.56, S, 8.11%.

### Preparation of Ag_12_bpy-CH_3_·O_2_ single crystals

A single crystal of Ag_12_bpy-CH_3_ was evacuated to a pressure of 10^−3^–10^−4^ Pa. Next, pure pressurized O_2_ was backfilled at 77 K through the open end of the capillary until the pressure reached 30 kPa. Subsequently, the open end was sealed in situ to maintain the crystal under high pure O_2_ pressure surroundings, producing Ag_12_bpy-CH_3_·O_2_, which was subjected to SCXRD analysis at 80 K.

### Preparation of Ag_12_bpy-NH_2_/CH_3_ crystals

Crystals of Ag_12_bpy-NH_2_/CH_3_ were prepared similar to Ag_12_bpy-NH_2_, except the mixture of bpy-NH_2_ and bpy-CH_3_ were added into the reaction solution simultaneously. Different ratios of bpy-NH_2_ to bpy-CH_3_ were attempted according to the relative intensity of two emission peaks. The optimized ratio of bpy-NH_2_:bpy-CH_3_ = 1:100 was determined by ^1^H NMR (Supplementary Fig. 22).

### Crystallographic data collection and refinement of the structure

SCXRD measurements of Ag_12_bpy-NH_2_ and Ag_12_bpy-CH_3_ were performed at 150 K and measurements of Ag_12_bpy-CH_3_·O_2_ were performed at 80 K on a Rigaku XtaLAB Pro diffractometers with Cu-Kα radiation (λ = 1.54184 Å). Data collection and reduction were performed using the program CrysAlisPro^51,52^. All the structures were solved with direct methods (SHELXS)^53^ and refined by full-matrix least squares on *F*^*2*^ using OLEX2^54^, which utilizes the SHELXL-2015 module^55^. All atoms were refined anisotropically, and hydrogen atoms were placed in calculated positions with idealized geometries and assigned fixed isotropic displacement parameters. Detailed information about the X-ray crystal data, intensity collection procedure and refinement results for Ag_12_bpy-NH_2_, Ag_12_bpy-CH_3_, and Ag_12_bpy-CH_3_·O_2_ is summarized in Supplementary Table 2.

### Calculation of void space

The fraction of void space was calculated from the X-ray structural data of Ag_12_bpy-NH_2_ by PLATON/VOID: The unit cell was filled with the atoms from the structural model, and each specific atom was assigned its respective van der Waals radius. A grid search generated a list of grid points with a minimum distance of 1.2 Å from the nearest van der Waals surface. This list of grid points was then used to produce a new list of grid points that makes up the solvent accessible areas. For the sets of grid points, the centre of gravity and volume of the void were calculated. The overall solvent accessible volume was calculated, along with the volume and centre of gravity of individual ‘voids’.

### Quantum chemical calculations

Density functional theory (DFT) calculations were performed in Gaussian 16^56^ using the PBE0 hybrid functional (mixes the Perdew–Burke–Ernzerhof^57^ and Hartree–Fock exchange energy in a set 3:1 ratio) with the 6-31 G* basis set for H, B, C, N, O, F, and S atoms^58,59^ and LanL2DZ effective core potentials for Ag atoms^60,61^. The single-crystal structure was chosen as the initial guess for ground-state geometry optimization, and all reported stationary points were verified as true minima by the absence of negative eigenvalues in the vibrational frequency analysis. The calculated absorption spectra were obtained from GaussSum 2.1^62^. Hirshfeld population analysis was conducted by Multiwfn 3.4^63^. Considering the ISC occurs at the excited states, the time-dependent DFT calculations were further carried out with the B3LYP/def2-SVP level based on the optimized structures. The spin-orbit coupling (SOC) matrix elements for the four systems were computed using the spin-orbit mean-field (SOMF) approach in the B3LYP/def2-SVP level in the ORCA 4.1.2 package^64^. The resolution of the identity (RI) approximation was used during the SOC calculations with the RIJONX flag to speed up the self-consistent field (SCF) convergence during each step. The auxiliary basis sets used with the RI approximation are built automatically by ORCA^65,66^. Over 3000 basis functions were included to create the molecular orbitals, and a typical calculation of 20 singlet and 20 triplet roots were calculated considering the molecules are extremely large (>300 atoms for each).

### Luminescence measurements

All solid-state Ag_12_bpy-NH_2_, Ag_12_bpy-CH_3_, and Ag_12_bpy-NH_2_/CH_3_ (1:100) samples were evacuated before the collection of luminescence spectra, in addition to solvent-dependent luminescence measurements in Supplementary Fig. 12. Steady-state photoluminescence (PL) spectroscopy, three-dimensional excitation-emission matrix (3D-EEM) luminescence spectroscopy, and emission decay spectroscopy were performed on a HORIBA instrument.

### Oxygen-sensing measurements

The Ag_12_bpy-NH_2_ samples were placed in a holder in a gas absorption analyser (BEL-max physisorption analyser, MicrotracBEL Belsorp Max with an ultimate vacuum of 6.7 × 10^−7^ Pa) to precisely control the O_2_ pressure at nearly anaerobic conditions, and this apparatus was connected to a spectrofluorometer (HORIBA FluoroLog-3) by an optical fibre to obtain a luminescence signal readout (see Supplementary Fig. 13). For the range from 20 ppm to air, standard mixed gases of O_2_ and N_2_ from Henan Yuanzheng Special Gas Development Co., Ltd., were used.

## Supplementary information

Supplementary Information

Peer Review File

## Data Availability

Data supporting the findings of this manuscript are available from the corresponding authors upon reasonable request. The X-ray crystallographic coordinates for structures reported in this article have been deposited at the Cambridge Crystallographic Data Centre (CCDC) under deposition number CCDC: 1963529 (Ag_12_bpy-NH_2_), 1963530 (Ag_12_bpy-CH_3_), 1963528 (Ag_12_bpy-CH_3_·O_2_).
