# Supplementary material for: Ligand engineering to achieve enhanced ratiometric oxygen sensing in a silver cluster-based metal-organic framework
Source: Nat Commun. 2020 Jul 22;11:3678. doi: 10.1038/s41467-020-17200-w (PMC7376137; doi:10.1038/s41467-020-17200-w)
Supplement: Supplementary file 1 — Supplementary Information [file 41467_2020_17200_MOESM1_ESM.pdf]

Ligand engineering to achieve enhanced ratiometric oxygen sensing in a silver cluster-based metal-organic framework

Xi-Yan Dong[1,2,†], Yubing Si,[1,†] Jin-Sen Yang,[1] Chong Zhang,[1] Zhen Han,[1] Peng Luo,[1] Zhao-Yang Wang,[1] Shuang-Quan Zang[1,*] and Thomas C. W. Mak[1,3]

[1]*Green Catalysis Center, and College of Chemistry, Zhengzhou University, Zhengzhou 450001, China*

[2]*College of Chemistry and Chemical Engineering, Henan Polytechnic University, Jiaozuo 454003, China.*

[3]*Department of Chemistry, The Chinese University of Hong Kong, Shatin, New Territories, Hong Kong SAR. China*

*E-mail: zangsqzg@zzu.edu.cn

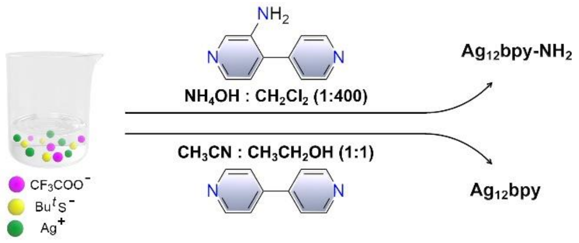

Supplementary Figure 1. The synthesis method of Ag_{12}bpy and Ag_{12}bpy-NH_2.

During the synthesis of Ag_{12}bpy-NH_2, the solvent was changed to CH_2Cl_2. Besides, since the slight basicity of bpy-NH_2 easily resulted in unknown precipitated solids, the presence of small quantity of ammonium hydroxide is also indispensable.

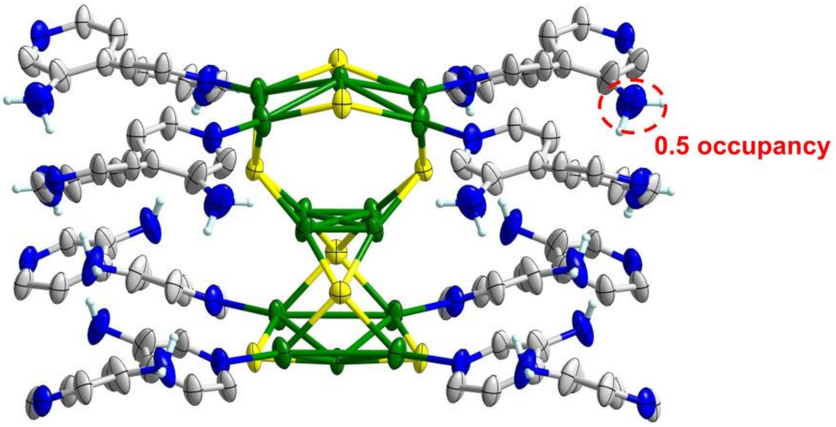

Supplementary Figure 2. Structure of $Ag_{12}(S)_8$(bpy-NH_2)$_8$ moiety in Ag_{12}bpy-NH_2 depicted with thermal ellipsoids shown at 50%. Perspective view of the $Ag_{12}S_8$ cluster node coordinated with eight bpy-NH_2 linkers in **Ag_{12}bpy-NH_2**. Each −NH_2 group shows 0.5 occupancy, resulting in two −NH_2 groups on two pyridine rings of each linker. Colour codes: green = silver; yellow = sulfur; grey = carbon; blue = nitrogen; turquoise = hydrogen.

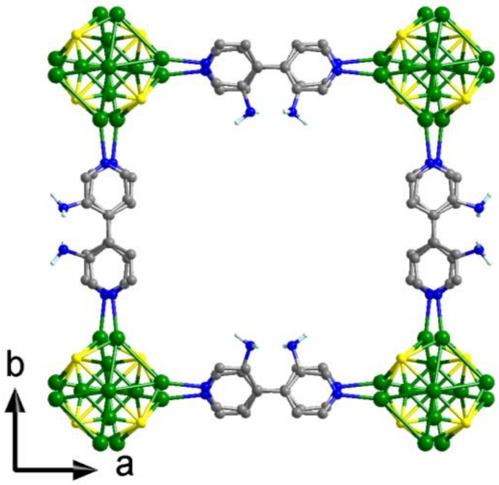

Supplementary Figure 3. Window of Ag_{12}bpy-NH_2 viewed along the *c* axis. The bpy-NH_2 linkers serve as edges and Ag–S clusters serve as vertices. Only one $-NH_2$ group is shown on the bpy-NH_2 linker.

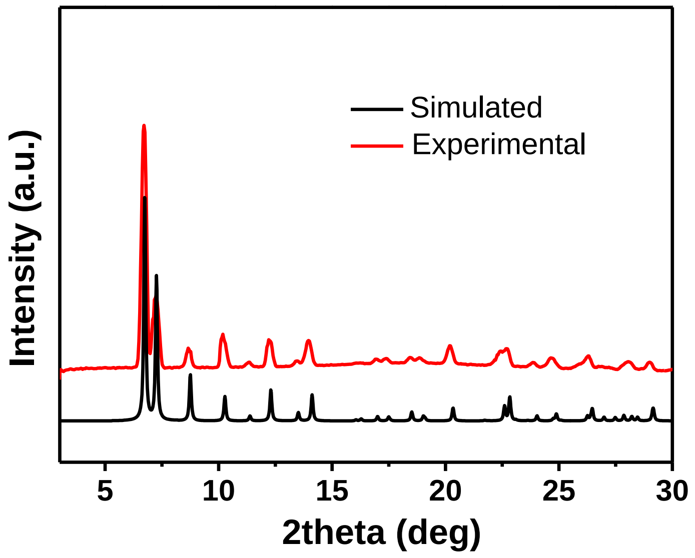

Supplementary Figure 4. PXRD patterns. The stimulated (black line) and as-synthesized (red line) **Ag_{12}bpy-NH_2** samples.

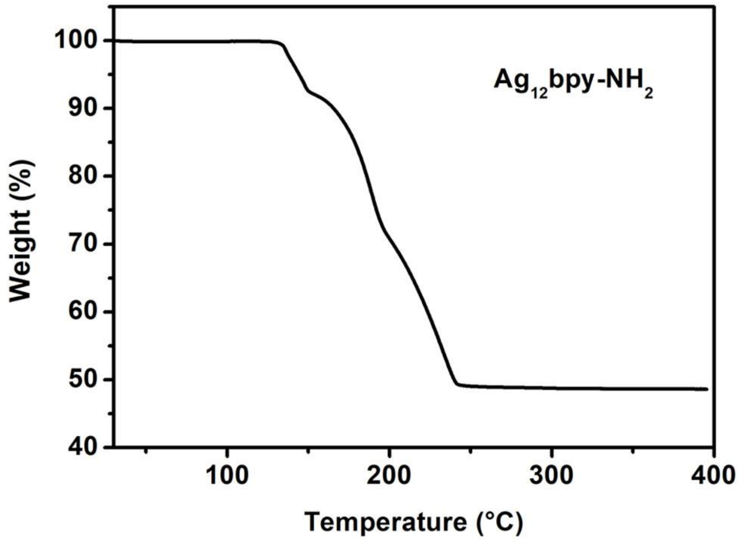

Supplementary Figure 5. TG plot. The as-synthesized **Ag_{12}bpy-NH_2** samples are measured in air.

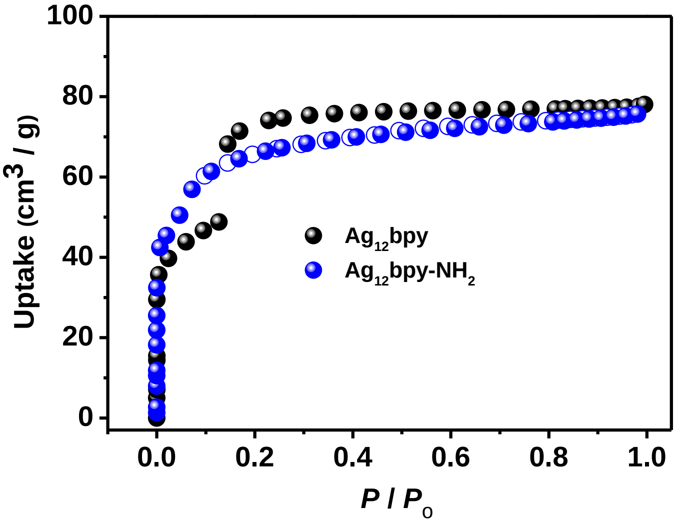

Supplementary Figure 6. Adsorption isotherms. Comparison of N_2 adsorption isotherms of **Ag_{12}bpy** (black line) and adsorption/desorption (blue solid/empty line) isotherms **Ag_{12}bpy-NH_2** at 77 K.

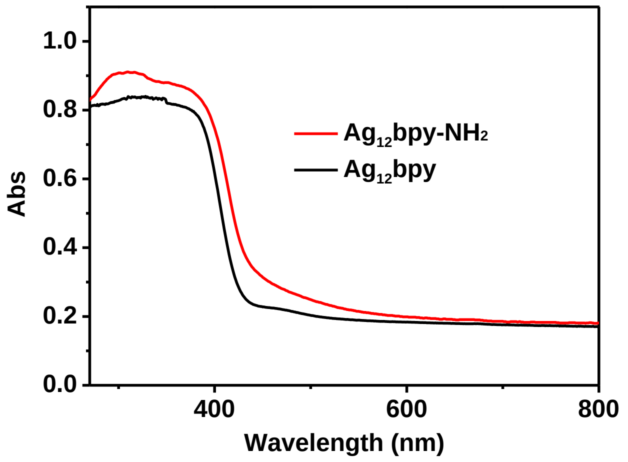

Supplementary Figure 7. UV-vis diffuse reflectance spectra. The solid-state **Ag_{12}bpy-NH_2** and **Ag_{12}bpy** samples are measured under ambient conditions.

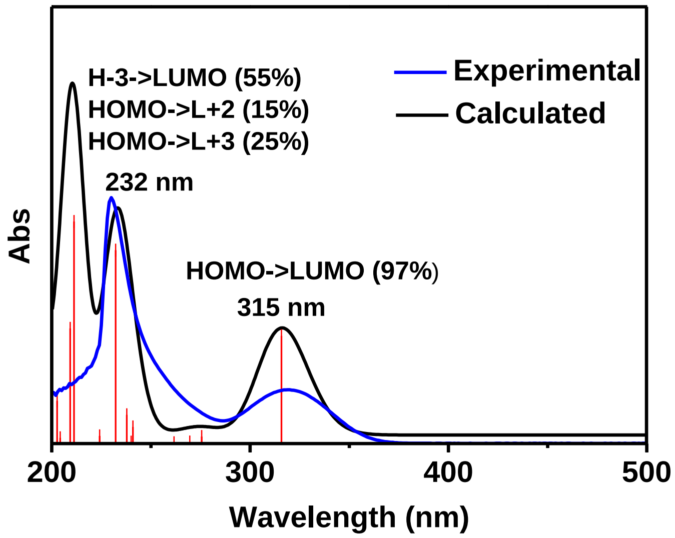

Supplementary Figure 8. Calculated and experimental optical absorption spectra of linker bpy-NH_2 (in CH_2Cl_2). Red sticks show the individual transitions (delta-function-like peaks showing the relative oscillator strengths).

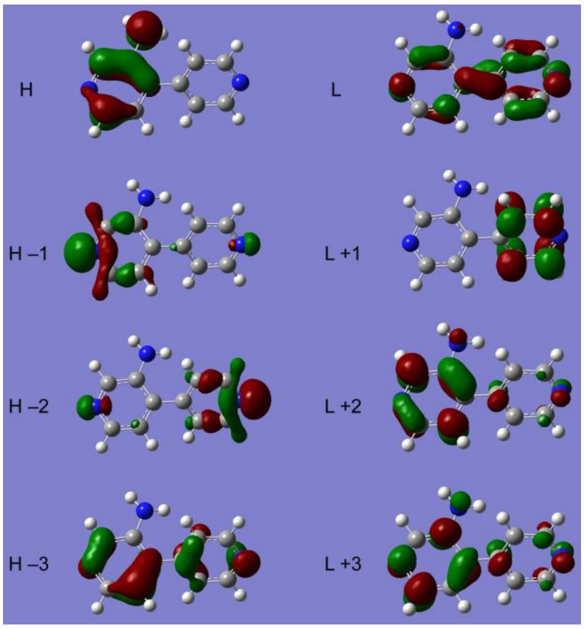

Supplementary Figure 9. Selected frontier MO representations for bpy-NH_2 linker. (HOMO=H, LUMO=L).

As shown in Supplementary Figs. 8 and 9, the transitions at approximately 315 nm mainly involve the HOMO-LUMO transition (95%), corresponding to a mixture of n-π* and π-π* transitions.

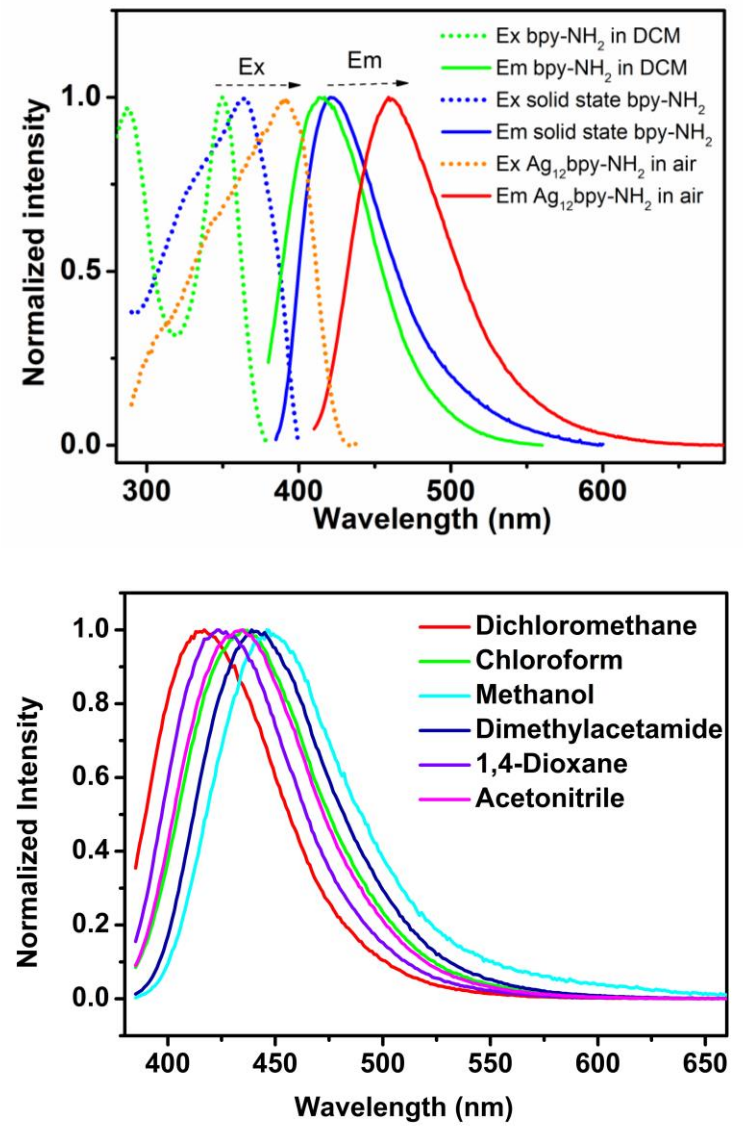

Supplementary Figure 10. Photoluminescence spectra. (top) Comparison of excitation (dashed line) and normalized emission (solid line) spectra among bpy-NH_2 in DCM (10^{-5} M), solid-state bpy-NH_2 and **Ag_{12}bpy-NH_2** in air. The redshift of the ligand-based blue emission from 420 nm to 460 nm after coordination to the Ag atom in **Ag_{12}bpy-NH_2**. (bottom) Normalized emission spectra of bpy-NH_2 in different solvents (5.5×10^{-4} M).

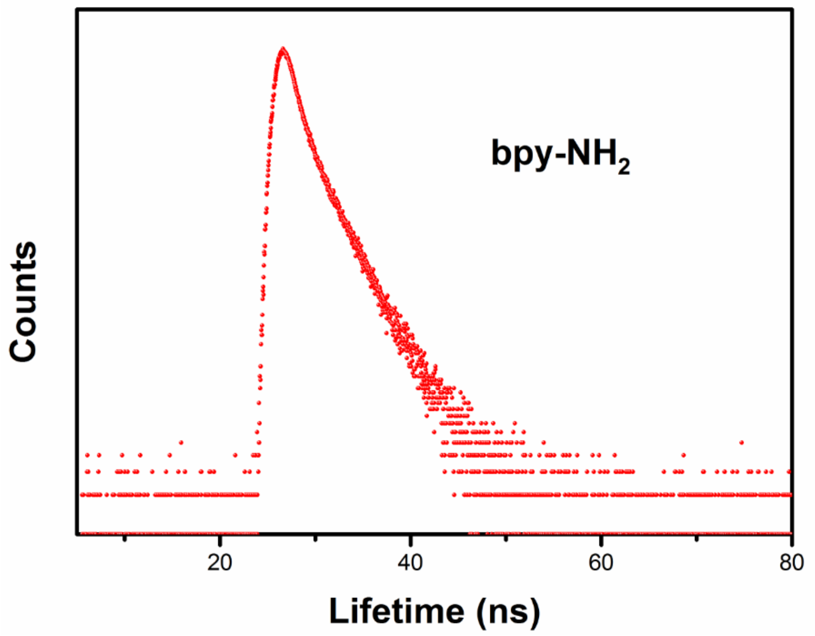

Supplementary Figure 11. Emission lifetime. Decay traces of solid-state bpy-NH_2. at room temperature.

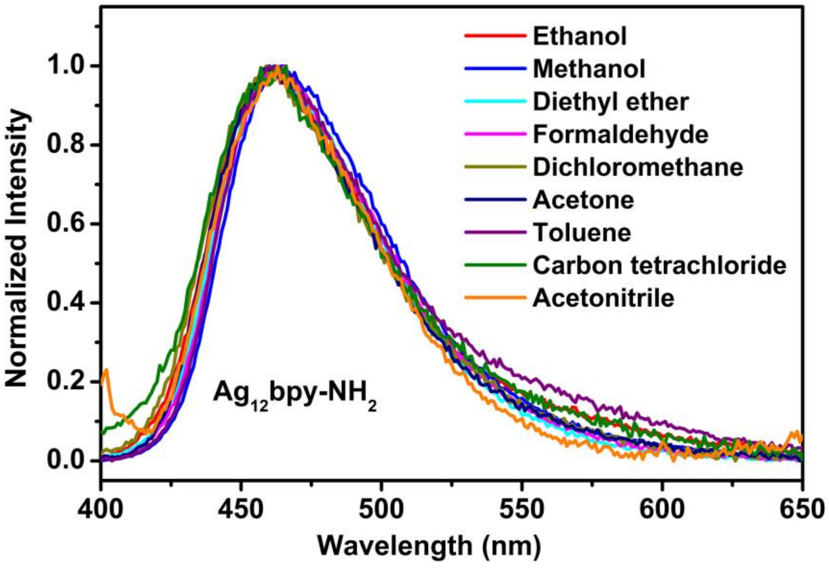

Supplementary Figure 12. Emission spectra. Normalized emission spectra of **Ag_{12}bpy-NH_2** with a small amount of organic solvent. The 460-nm emission spectra are nearly independent of the solvent, demonstrating that the emission could result from the $^1(\pi, \pi^*)$ singlet state or from a mixture of the $^1(\pi, \pi^*)/(n, \pi^*)$ singlet states.

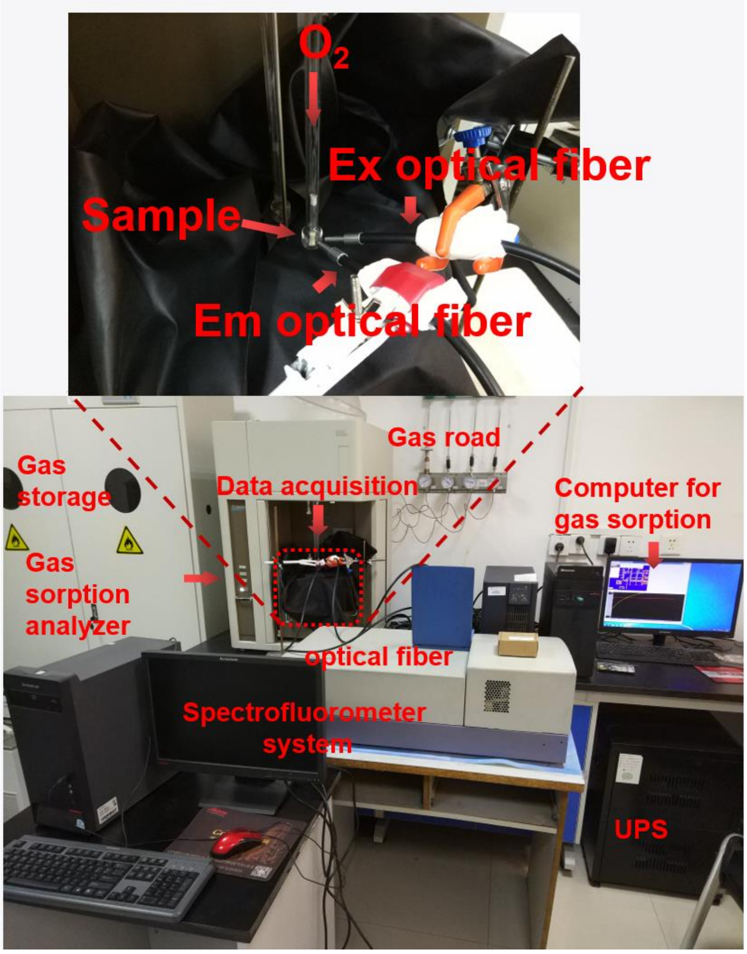

Supplementary Figure 13. Luminescence measurement system for ultra-low concentration of oxygen. Image of the homemade high-vacuum cell used for monitoring the emission in the presence of an ultra-low concentration of oxygen. Ex=excitation; Em=emission.

In-situ oxygen responses of photoluminescence were carried out with the set-up that combines fluorescence signal collection and oxygen partial pressure control. The fluorescence spectra were collected through a Horiba Scientific FluoroMax-3 spectrofluorometer equipped with an FM4-3000 Fiber-Optic Adaptor, while the oxygen partial pressure was controlled by a 3H-2000Ps1 analysis instrument. The excitation light source was extracted from a Xe lamp of the spectrophotometer through the first fiber bundle of the Fiber-Optic Adaptor. The emission signals were collected by the detector of the spectrophotometer through the second fiber bundle. The quartz container with fresh samples (~100 mg) was connected to the gas control instrument. Before injecting O_2, the tube was evacuated for 12 hours until the pressure is lower than 1.0×10^{-4} pa. The excitation fiber bundle and the emission fiber bundle were pointed at an angle of ~30 degrees towards the quartz container. Both bundles were covered by a light-shielding cloth. In addition, before each measurement, a slight amount of adjustment was performed to the bundles to obtain the maximum signal.

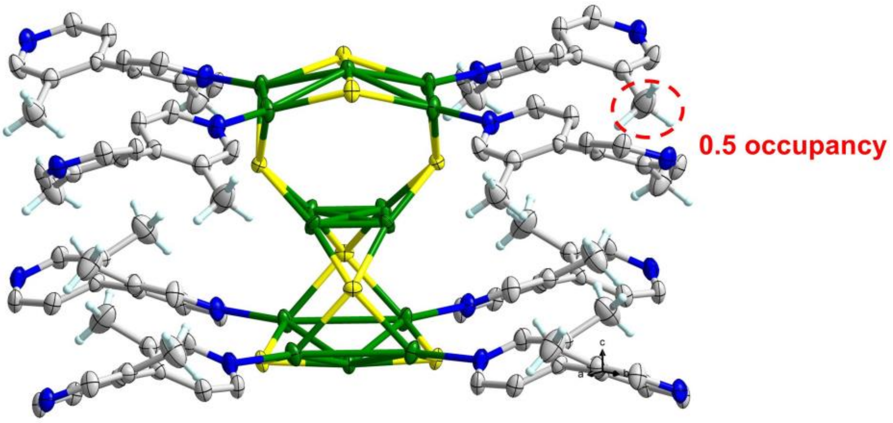

Supplementary Figure 14. Structure of $Ag_{12}(S)_8(bpy\text{-}CH_3)_8$ moiety in Ag_{12}bpy-CH_3 depicted with thermal ellipsoids shown at 50%.
Perspective view of the $Ag_{12}S_8$ cluster node coordinated with eight bpy-CH_3 linkers in **Ag_{12}bpy-CH_3**. Each −CH_3 group shows 0.5 occupancy, resulting in two -NH_2 groups on two pyridine rings of each linker. Colour codes: green = silver; yellow = sulfur; grey = carbon; blue = nitrogen; turquoise = hydrogen.

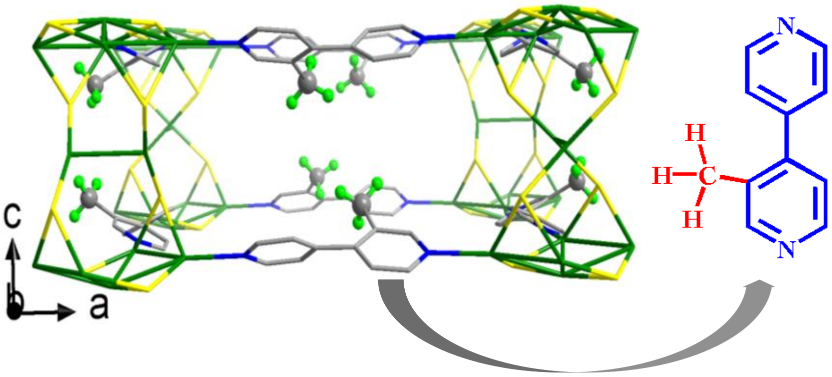

Supplementary Figure 15. Channels of Ag_{12}bpy-CH_3 viewed along the *b* axis. The CH_3 protruding inwards into the channels; the structure of the bpy-CH_3 linker.

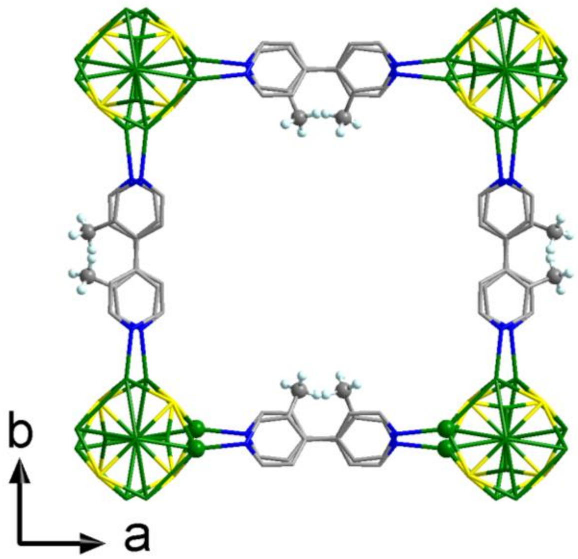

Supplementary Figure 16. Square window in one double-layer of Ag_{12}bpy-CH_3 viewed along the *c* axis. bpy-CH_3 linkers serve as edges and Ag–S clusters serve as vertices. Only one −CH_3 group is shown on each bpy-CH_3 linker.

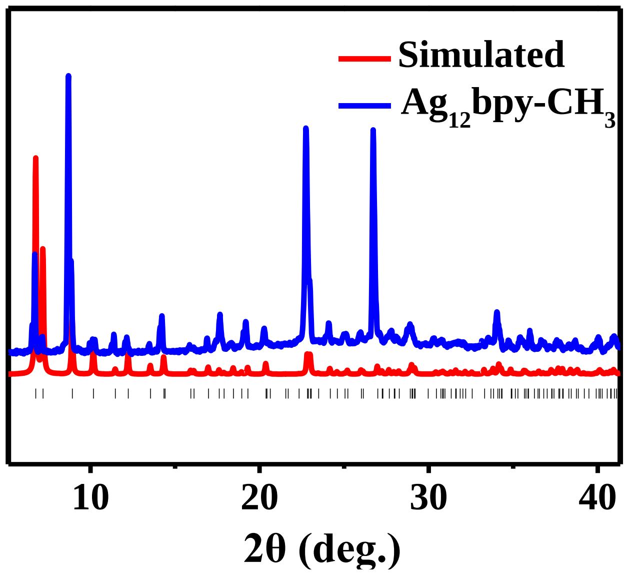

Supplementary Figure 17. PXRD patterns. The simulated PXRD pattens from the single-crystal data of **Ag_{12}bpy-CH_3** and the measured PXRD pattens of as-prepared **Ag_{12}bpy-CH_3** samples.

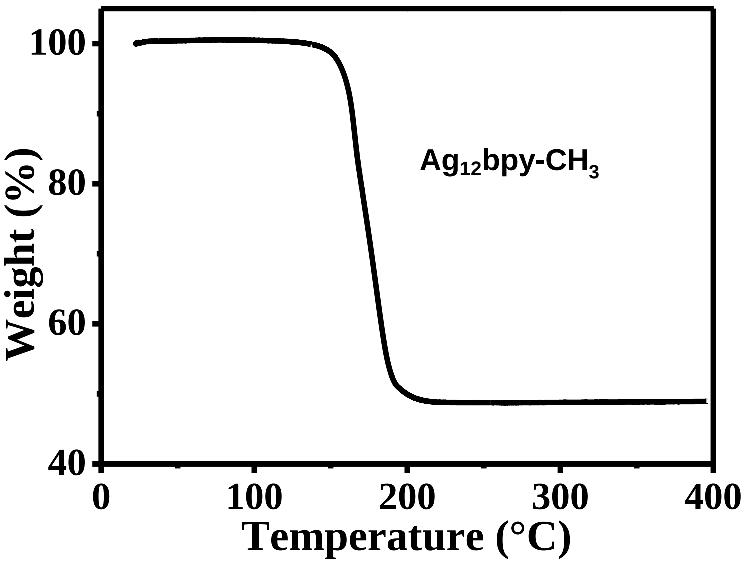

Supplementary Figure 18. TG plot. The crystalline **Ag_{12}bpy-CH_3** samples are measured under oxygen conditions.

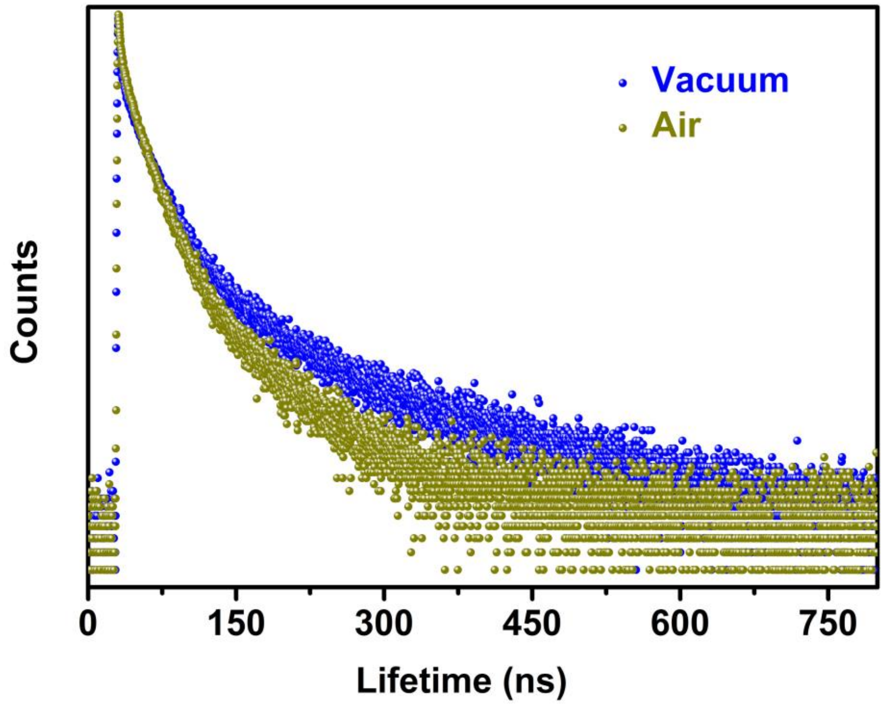

Supplementary Figure 19. Emission lifetimes. Time-resolved decay traces of **Ag_{12}bpy-CH_3** monitored at 500 nm at 293 K in air and under vacuum, respectively.

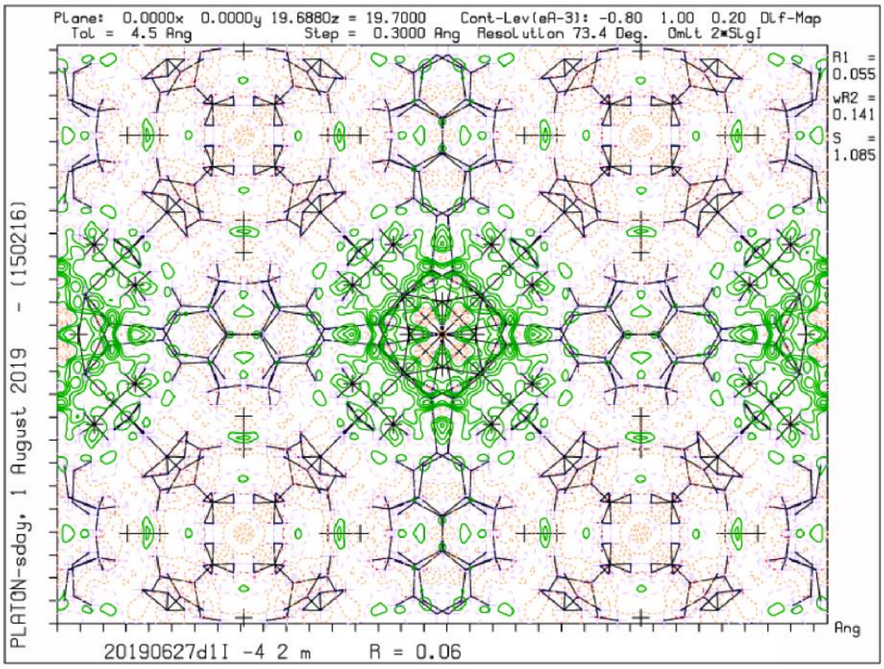

Supplementary Figure 20. Difference Fourier maps of Ag_{12}bpy-CH_3·O_2 in the crystallographic (0 0 1) plane.

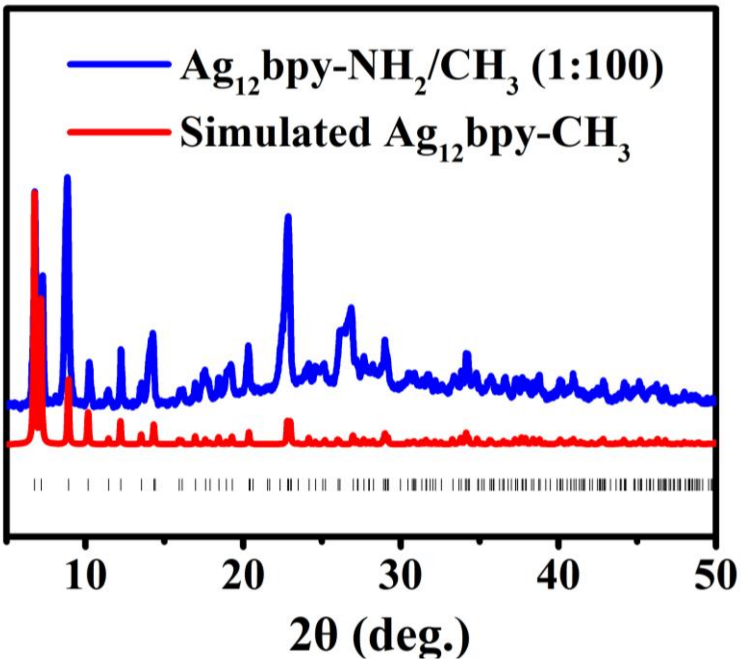

Supplementary Figure 21. PXRD patterns. The simulated PXRD pattens from the single-crystal data of **Ag_{12}bpy-CH_3** and the measured PXRD pattens of as-prepared **Ag_{12}bpy-NH_2/CH_3 (1: 100)**.

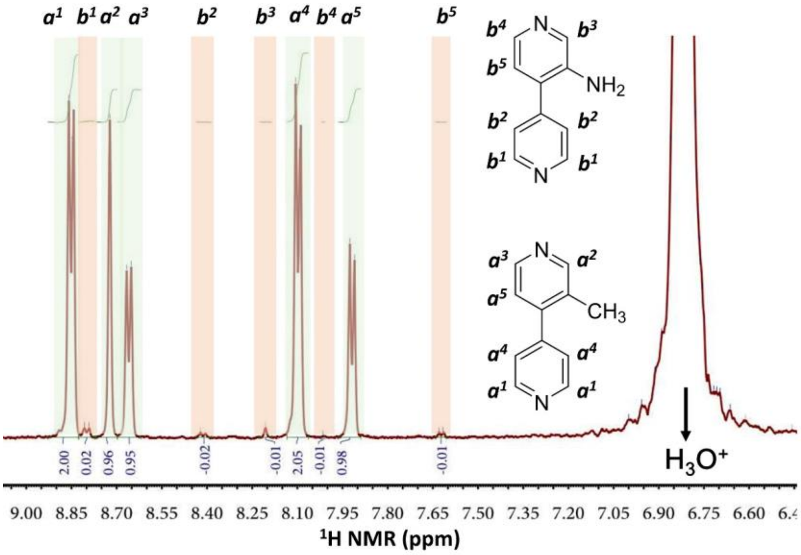

Supplementary Figure 22. ^{1}H-NMR spectra of as-prepared Ag_{12}bpy-NH_2/CH_3 (1: 100) samples. The crystal samples are digested in a DCl/DMSO-d6 mixture. H_3O^+ was provided by the H_2O and HCl contained in DCl.

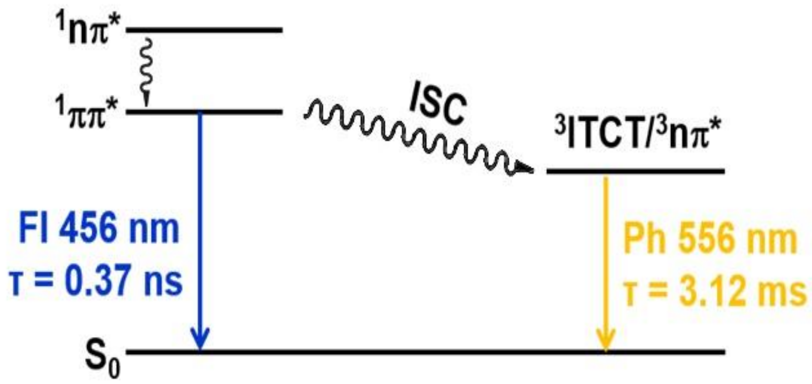

Supplementary Figure 23. Schematic of the dual Fl-Ph emission of Ag_{12}bpy-NH_2. The blue fluorescence (Fl) component at approximately 456 nm with a lifetime of 0.37 ns possibly originates from the mixed ^{1}(n, π*) and ^{1}(π, π*) excited singlet states; the yellow phosphorescence (Ph) component with a lifetime of 3.12 ms might originate from the mixed ^{3}ITCT and ^{3}(n, π*) triplet states. ISC from ^{1}(π, π*) to ^{3}ITCT /^{3}(n, π*) is activated.

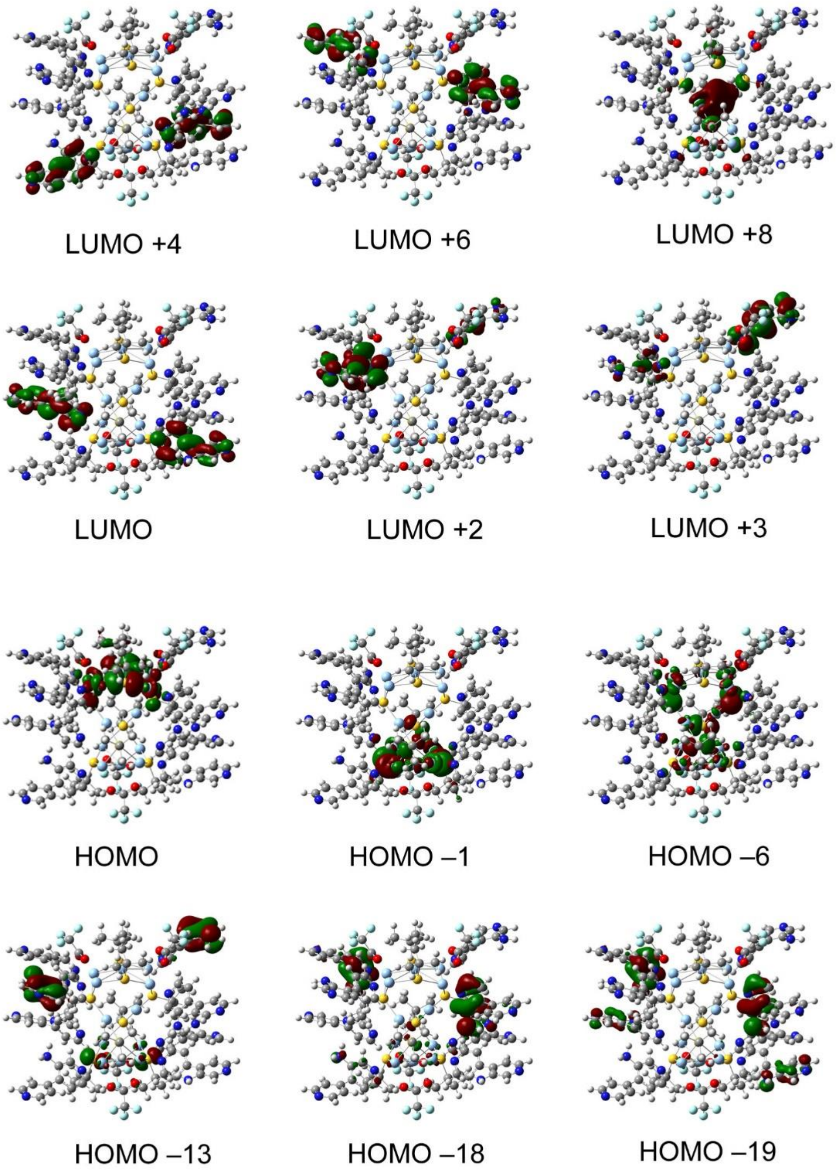

Supplementary Figure 24. Selected frontier MO representations for Ag_{12}bpy-NH_2. The $Ag_{12}(SBu^t)_8(CF_3COO)_4(bpy\text{-}NH_2)_8$ model is selected from singe crystal data.

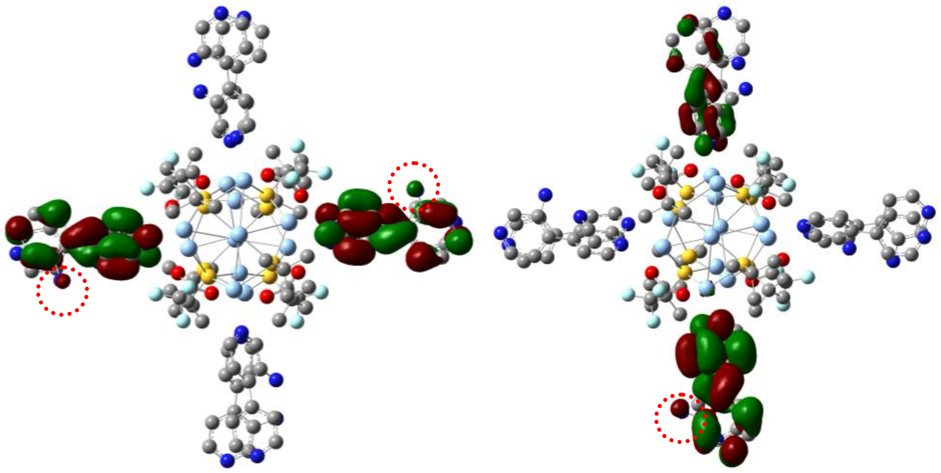

Supplementary Figure 25. LUMO and LUMO+2 configurations for Ag_{12}bpy-NH_2. These orbitals bear a hybrid configuration (n, π*) (LUMO = L) for the $Ag_{12}(SBu^t)_8(CF_3COO)_4(bpy\text{-}NH_2)_8$ model.

Supplementary Table 1. Hirshfeld percentage of orbital compositions in Ag_{12}bpy-NH_2. Comparison of Hirshfeld percentage (B3LYP/(LanL2DZ/6-31G*) level) of orbital compositions (Com.) of frontier MOs in **Ag_{12}bpy-NH_2**. Ag refers to all twelve silver atoms in the core. S and O refer to the coordinated atoms of eight –SBu^t and four CF_3COO^- groups, respectively. Bpy refers to the eight 4,4'-bipyridine moieties of a cluster model. NH_2 refers to the substituted group on the bpy-NH_2 linker. (H = HOMO, L = LUMO).

	Ag	S	bpy	NH_2
Orb#	Com.(%)	Com.(%)	Com.(%)	Com.(%)
H-19	<3.0	<3.0	<5.0	26.7
H-18	<5.0	3.6	<3.0	24.7
H-13	5.4	9.5	36.7	11.9
H-1	37.1	48.5	<3.0	<1.0
HOMO	36.5	47.6	<3.0	<1.0
LUMO	<3.0	<3.0	83.4	<1.0
L+1	<3.0	<1.0	84.8	<1.0
L+2	<3.0	<1.0	64.4	<3.0
L+3	<3.0	<1.0	90.1	<3.0
L+6	<3.0	<3.0	80.6	<1.0
L+7	<3.0	<3.0	80.6	<1.0
L+8	54.4	23.1	<1.0	<1.0

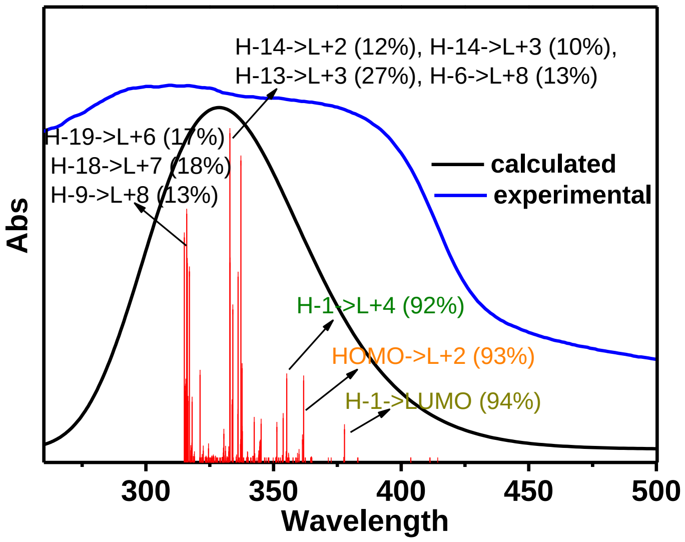

Supplementary Figure 26. Calculated optical absorption spectra of Ag_{12}bpy-NH_2. Calculated optical absorption spectra of the model, e.g., $Ag_{12}(SBu^t)_8(CF_3COO)_4(bpy\text{-}NH_2)_8$, and experimental solid-state UV-vis diffuse reflectance spectra of **Ag_{12}bpy-NH_2**. The red vertical bars represent vertical $S_n \leftarrow S_0$ excitations calculated for the optimized ground-state geometries. The bar sizes are proportional to the calculated oscillator strengths.

The calculated molecular orbitals of **Ag_{12}bpy-NH_2**: the highest occupied molecular orbital (HOMO) is composed of 47.6% S and 36.5% Ag, with a small contribution from bpy and -NH_2, while the contribution percentage of occupied orbitals to the main electronic transitions have significantly increased. For example, HOMO-13 consists of 36.7% bpy, 11.9% lone pair of NH_2, 9.5% S and 5.6% Ag, and HOMO-18 and HOMO-19 consist of 24.7% and 26.7% contributions from the lone pair of the amine. The lowest unoccupied molecular orbital (LUMO) contains a large contribution from the π^* orbitals of the two pyridines (83.4%), and LUMO+2, +3, +6, +7 are also dominated by π^* orbitals; however, LUMO+8 is composed of 54.4% Ag and 23.1% S. Therefore, the transitions with lower energies, namely H-1→LUMO, HOMO→L+2, and H-1→L+4, are related to ITCT and S/Ag → bpy; the HOMO→L+3 transition includes a mixture of ITCT, $n \rightarrow \pi^*$ and $\pi \rightarrow \pi^*$ transitions; and H-18 →L+7 and H-19→L+6 mainly involve $n \rightarrow \pi^*$ transitions (Supplementary Figs. 24–26 and Supplementary Table 1).

Supplementary Table 2. Crystal data and structure refinement for **Ag_{12}bpy-NH_2**, **Ag_{12}bpy-CH_3** and **Ag_{12}bpy-CH_3·O_2**.

	**Ag_{12}bpy-NH_2**	**Ag_{12}bpy-CH_3**	**Ag_{12}bpy-CH_3·O_2**
CCDC number	1963529	1963530	1963528
Empirical formula	$C_{80}H_{108}Ag_{12}F_{12}N_{12}O_8S_8$	$C_{84}H_{112}Ag_{12}F_{12}N_8O_8S_8$	$C_{80}H_{104}Ag_{12}F_{12}N_8O_{9.6}S_8$
Formula weight	3144.70	3140.73	3158.27
Temperature / K	150 K	150 K	81 K
Crystal system	tetragonal	tetragonal	tetragonal
Space group	I-42m	I-42m	I-42m
a /Å	17.31644(12)	17.37014(19)	17.32750(10)
b /Å	17.31644(12)	17.37014(19)	17.32750(10)
c /Å	19.4319(4)	19.7296(4)	19.6880(2)
α /°	90	90	90
β /°	90	90	90
γ /°	90	90	90
Volume /$Å^3$	5826.82(13)	5952.86(18)	5911.17(9)
Z	2	2	2
ρ_{calc} g/cm^3	1..792	1.752	1.774
μ /mm^{-1}	17.708	2.132	17.460
F(000)	3072.0	3072.0	3082.0
Crystal size/mm^3	0.1× 0.12× 0.15	0.12× 0.12× 0.2	0.1× 0.12× 0.12
Radiation	Cu Kα (λ = 1.54184)	Mo Kα (λ = 0.71073)	Cu Kα (λ = 1.54184)
2Θ range for data collection /°	6.838 to 134.678	4.69 to 57.914	6.796 to 146.77
Index ranges	$-20 \le h \le 14$, $-20 \le k \le 20$, $-22 \le l \le 22$	$-22 \le h \le 22$, $-19 \le k \le 23$, $-26 \le l \le 25$	$-21 \le h \le 18$, $-14 \le k \le 21$, $-16 \le l \le 24$
Reflections collected	11324	25636	7321
Independent reflections	2711 [R_{int} = 0.0339, R_{sigma} = 0.0274]	3760 [R_{int} = 0.0309, R_{sigma} = 0.0193]	2851 [R_{int} = 0.0613, R_{sigma} = 0.0584]
Data/restraints/parameters	2711/39/192	3760/48/187	2851/31/186
Goodness-of-fit on F^2	1.099	1.024	1.104
Final *R* indexes [I>=2σ (I)]	R_1 = 0.0331, wR_2 = 0.0810	R_1 = 0.0184, wR_2= 0.0397	R_1 = 0.0560, wR_2 = 0.1440
Final *R* indexes [all data]	R_1 = 0.0342, wR_2 = 0.0814	R_1 = 0.0203, wR_2 = 0.0403	R_1 = 0.0575, wR_2 = 0.1452
Largest diff. peak/hole / e $Å^{-3}$	1.08/–0.54	0.43/–0.34	1.74/–0.85
Flack parameter	-0.036(12)	0.019(7)	0.030(19)
