# Supplementary material for: Ligand engineering to achieve enhanced ratiometric oxygen sensing in a silver cluster-based metal-organic framework
Source: Nat Commun. 2020 Jul 22;11:3678. doi: 10.1038/s41467-020-17200-w (PMC7376137; doi:10.1038/s41467-020-17200-w)
Supplement: Supplementary file 2 — Peer Review File [file 41467_2020_17200_MOESM2_ESM.docx]

Peer Review Information

**Journal:** Nature Communications

**Manuscript title:** Ligand engineering to achieve record-breaking ratiometric oxygen sensing in a silver cluster-based metal-organic framework

**Corresponding author name(s):** Dr Shugan-Quan Zang

**Editorial notes:**

**none**

**Reviewer comments & decisions:**

**Reviewer comments, first version:**

Reviewer #1 (Remarks to the Author):

Dong et al. reports a ratiometric oxygen sensor based on silver-chalcogenolate-cluster-based metal-organic frameworks (SCC-MOFs) using ligand engineering strategy to achieve a dual emission. MOFs with metal clusters as nodes are a new type of porous luminescent functional material. The interesting dual-emission of MOFs that originates from the subtly balanced excited states is attracting in a diversity of sensing applications. The authors rationally introduced amine groups with lone-pair electrons on the linkers to modulate spin-orbit coupling and increase the rate of intersystem crossing. As a result, besides the additional blue Fl component, the emission lifetime of Ph component was prolonged 15,000 times, which not only led to dual fluorescent-phosphorescent emission but also significantly enhanced sensitivity to oxygen molecules. A 100-nm gap in wavelength and 850,000-fold difference in lifetime between Fl component and Ph component enable the negligible interference. This is very scarce in luminescent MOFs, which also displayed unique advantages in tuning luminescence based on unique emissive mechanism of metal cluster-based MOFs. This work represents a big breakthrough from this lab again. I would like to recommend the publication on Nature Communications. Some minor revisions are needed.

1. A brief introduction of luminescence modulation of dual-emissive MOFs should be added in introduction section. Thus, readers can easily know the research background and the novelty of this work.
2. Metal clusters are commonly flexible and their structures are easily affected by reaction conditions. The presented isostructural metal cluster-MOFs are the first cases, which also provided the excellent models for researching the emissive mechanism. The different synthesis between Ag12bpy-NH2 and Ag12bpy is illustrated in Figure S1. Can the possible effects of synthesis method be briefly discussed?
3. The homemade high-vacuum cell is critical in the measurements of the oxygen pressure-dependent emission in the range of ppm level. It is indeed difficult to measure the emission variation of luminescent materials induced by ppm-level gas oxygen molecules. Therefore, please provide more details of the homemade emission system operating under ultra-high vacuum. It could be used in other volatile compounds sensing more precisely.
4. Figure 6 can be included in Supplementary Information.


Reviewer #2 (Remarks to the Author):

This manuscript reports ligand engineering to achieve ratiometric oxygen sensing in a silver cluster-based metal-organic framework by modifying a previously reported silver cluster-based metal-organic framework. By introducing -NH2 groups on the linkers of the MOF, authors could achieve a dual FL-Ph emission from the same linker chromophore and quantified oxygen with excellent sensitivity. Furthermore, authors used a substituent-mediated strategy (-CH3 and mixed -NH2 and -CH3 roups) to interfere with the dynamic quenching process of O2 and extended the sensing concentration range.
Overall, this nice piece of systematic work of oxygen sensing using porous framework materials. Experiments were carried out carefully and the manuscript is well written. I don’t see any obvious technical error in the manuscript. I recommend publication of the manuscript in NatureCommun after following minor revisions.

Fig. 3 presented is more artistic than informative. Authors should put the figure in the SI with better close views with original pictures for give more clear idea about the set up.
Texts in Fig 4c and 4d are not clear. Need to improve.
Mixed-linker crystal of Ag12bpy-NH2/CH3 (with a bpy-NH2: bpy-CH3 ratio of 1:100) was prepared to check the oxygen-sensing performance. Is there any logic for this ratio or it’s just one random ration. Should explain.

**Author rebuttal, first version:**

Reviewer #1 (Remarks to the Author):

Dong et al. reports a ratiometric oxygen sensor based on silver-chalcogenolate-cluster-based metal-organic frameworks (SCC-MOFs) using ligand engineering strategy to achieve a dual emission. MOFs with metal clusters as nodes are a new type of porous luminescent functional material. The interesting dual-emission of MOFs that originates from the subtly balanced excited states is attracting in a diversity of sensing applications. The authors rationally introduced amine groups with lone-pair electrons on the linkers to modulate spin-orbit coupling and increase the rate of intersystem crossing. As a result, besides the additional blue Fl component, the emission lifetime of Ph component was prolonged 15,000 times, which not only led to dual fluorescent-phosphorescent emission but also significantly enhanced sensitivity to oxygen molecules. A 100-nm gap in wavelength and 850,000-fold difference in lifetime between Fl component and Ph component enable the negligible interference. *This is very scarce in luminescent MOFs, which also displayed unique advantages in tuning luminescence based on unique emissive mechanism of metal cluster-based MOFs*. *This work represents a big breakthrough from this lab again. I would like to recommend the publication on Nature Communications.* Some minor revisions are needed.

**Response:** Thank you very much for these positive comments and professional suggestions.

1. A brief introduction of luminescence modulation of dual-emissive MOFs should be added in introduction section. Thus, readers can easily know the research background and the novelty of this work.

**Response:** Thanks for the comments. As suggested by **Reviewer 1**, we have added the background of luminescence modulation of dual-emissive MOFs in the Introduction Section of the revised manuscript, and the changes are highlighted in yellow.

2. Metal clusters are commonly flexible and their structures are easily affected by reaction conditions. The presented isostructural metal cluster-MOFs are the first cases, which also provided the excellent models for researching the emissive mechanism. The different synthesis between Ag12bpy-NH2 and Ag12bpy is illustrated in Figure S1. Can the possible effects of synthesis method be briefly discussed?

**Response:** Thanks for the comments. As suggested by **Reviewer 1**, we have added the discussion of different synthesis method of Ag12bpy-NH2 and Ag12bpy in Supplementary Scheme 1. The changes are highlighted in yellow.

3. The homemade high-vacuum cell is critical in the measurements of the oxygen pressure-dependent emission in the range of ppm level. It is indeed difficult to measure the emission variation of luminescent materials induced by ppm-level gas oxygen molecules. Therefore, please provide more details of the homemade emission system operating under

College of Chemistry, Zhengzhou University, Zhengzhou 450001, China	1

ultra-high vacuum. It could be used in other volatile compounds sensing more precisely. **Response:** Thanks for the comments. According to the suggestions of **Reviewer 1**, we have added more details of the homemade emission system under ultra-high vacuum, which are added as Supplementary Fig. 12 and highlighted in yellow in the Supplementary Information. Our measurement operation on this system is also applicable to other gases or volatile compounds.

|  | **化学学院**  **College of Chemistry** |
| --- | --- |

4. Figure 6 can be included in Supplementary Information.

**Response:** Thanks for the comments. As suggested by **Reviewer 1**, we have included original Figure 6 in the revised Supplementary Information as Supplementary Fig. 22. The changes are highlighted in yellow.

**Responses to Reviewer 2**

Reviewer #2 (Remarks to the Author):

This manuscript reports ligand engineering to achieve ratiometric oxygen sensing in a silver cluster-based metal-organic framework by modifying a previously reported silver cluster-based metal-organic framework. By introducing -NH2 groups on the linkers of the MOF, authors could achieve a dual FL-Ph emission from the same linker chromophore and quantified oxygen with excellent sensitivity. Furthermore, authors used a substituent-mediated strategy (-CH3 and mixed -NH2 and -CH3 groups) to interfere with the dynamic quenching process of O2 and extended the sensing concentration range. *Overall, this nice piece of systematic work of oxygen sensing using porous framework materials. Experiments were carried out carefully and the manuscript is well written. I don’t see any obvious technical error in the manuscript. I recommend publication of the manuscript in NatureCommun. after following minor revisions.*

**Response:** We appreciate **Reviewer 2**s positive evaluation.

Fig. 3 presented is more artistic than informative. Authors should put the figure in the SI with better close views with original pictures for give more clear idea about the set up.

**Response:** Thanks for the comments. As suggested by **Reviewer 2**, we have added original pictures of the set up and detailed explanations instead of the schematic diagram in the Supplementary Fig. 12 in the revised Supplementary Information. The changes are highlighted in yellow.

Texts in Fig 4c and 4d are not clear. Need to improve.

**Response:** Thanks for the comments. As suggested by **Reviewer 2**, we have carefully checked

and improved the texts in Fig 4c and 4d as well as the others parts of the manuscript.

Mixed-linker crystal of Ag12bpy-NH2/CH3 (with a bpy-NH2: bpy-CH3 ratio of 1:100) was prepared to check the oxygen-sensing performance. Is there any logic for this ratio or it’s just one random ration. Should explain.

**Response:** Thanks for the comments. The ratios of bpy-NH2 to bpy-CH3 in the mixed-linker crystals of Ag12bpy-NH2/CH3 were chosen according to the relative intensity of the two emission peaks. According to the comments of **Reviewer 2**, we have added more information about the ratio of bpy-NH2 to bpy-CH3 in the “Methods Section” of the revised manuscript, which are highlighted in yellow.

**Reviewer comments, second version:**

Reviewer #1 (Remarks to the Author):

The authors have addressed my previous queries, and I have no further comments.Publish as it is now.

**Author rebuttal, second version:**

**Reviewer #1 (Remarks to the Author):**

The authors have addressed my previous queries, and I have no further comments. Publish as it is now.

**Response:** Thanks very much for your kind review on our paper.

**Reviewer comments, third version:**

**Author rebuttal, third version:**
